# Lipotoxicity Downstream of α-Synuclein Imbalance: A Relevant Pathomechanism in Synucleinopathies?

**DOI:** 10.3390/biom12010040

**Published:** 2021-12-28

**Authors:** Arati Tripathi, Saranna Fanning, Ulf Dettmer

**Affiliations:** Ann Romney Center for Neurologic Diseases, Department of Neurology, Brigham and Women’s Hospital, Harvard Medical School, Boston, MA 02115, USA; sfanning2@bwh.harvard.edu

**Keywords:** alpha-synuclein, lipids, lipidopathy, Parkinson’s disease, Lewy body dementia, neurotoxicity, stearoyl-CoA desaturase

## Abstract

Neuronal loss in Parkinson’s disease and related brain diseases has been firmly linked to the abundant neuronal protein α-synuclein (αS). However, we have gained surprisingly little insight into how exactly αS exerts toxicity in these diseases. Hypotheses of proteotoxicity, disturbed vesicle trafficking, mitochondrial dysfunction and other toxicity mechanisms have been proposed, and it seems possible that a combination of different mechanisms may drive pathology. A toxicity mechanism that has caught increased attention in the recent years is αS-related lipotoxicity. Lipotoxicity typically occurs in a cell when fatty acids exceed the metabolic needs, triggering a flux into harmful pathways of non-oxidative metabolism. Genetic and experimental approaches have revealed a significant overlap between lipid storage disorders, most notably Gaucher’s disease, and synucleinopathies. There is accumulating evidence for lipid aberrations causing synuclein misfolding as well as for αS excess and misfolding causing lipid aberration. Does that mean the key problem in synucleinopathies is lipotoxicity, the accumulation of harmful lipid species or alteration in lipid equilibrium? Here, we review the existing literature in an attempt to get closer to an answer.

## 1. α-Synuclein and Synucleinopathies

α-Synuclein (αS) is an abundant protein of 140 residues present in all human neuron types. αS biology has attracted great interest given its association with a group of devastating neurodegenerative disorders known as synucleinopathies, the most prevalent of which is Parkinson’s disease (PD), followed by dementia with Lewy bodies (DLB) and multiple system atrophy (MSA). PD is a debilitating and progressive movement disorder that affects nearly 10 million people worldwide, and yet measures that prevent or slow its course are currently lacking. PD pathogenesis has been linked to increased αS expression levels by virtue of *SNCA* genetic locus duplication/triplication [1,2], variabilities in *SNCA*-promoter region REP1 [3,4], the *SNCA* 3′ region [4] or *SNCA* somatic copy number gains [5]. Single-point mutations in *SNCA* have also been identified in PD patients [6,7,8,9,10,11,12,13] and, together with duplication/triplication and other gene mutations of high penetrance, are commonly referred to as the familial form of PD (fPD). Moreover, Laperle et al. recently reported increased levels of soluble αS protein in dopaminergic neurons of patients suffering from young-onset PD (YOPD), defined by onset at <50 years [14]. These dopaminergic neurons bear no mutations in nine established PD genes: *EIFG1*, *PARK2*, *LRRK2, GBA, SNCA, PINK1, PARK7, VPS35*, and *ATP13A2*. No extra copies of *SNCA* were detected either, indicating a yet unknown cause for the increased αS levels. Although fPD cases account for less than 10% of all PD in the general population, abnormal accumulation of αS is present in virtually all patients with PD and related synucleinopathies. In the patient brain, αS-rich lesions can be found in neuronal somata (Lewy bodies, LBs) and in neurites (Lewy neurites, LNs). The exact nature of the αS accumulation/aggregation has been under debate, not least since a recent study described Lewy pathology as largely rich in lipids and membranous organelles, although ~20% of these LBs and LNs also contained large (5 nm) αS fibrils [15,16]. This was in contrast to the traditional characterization of LBs as consisting of mainly fibrillar αS aggregates [17]. While neuronal loss in PD and related brain diseases has been firmly linked to the abundant neuronal protein αS, we have gained surprisingly little insight into how exactly αS exerts toxicity in these diseases, eventually causing neurons to die. Diverse mechanisms including proteotoxicity, disturbed vesicle trafficking, mitochondrial dysfunction, reactive oxygen species, inflammation or a combination cascade of such mechanisms have been proposed. Related to the aforementioned debate about the nature of LBs, the mechanism of αS-related “lipotoxicity” has caught increasing attention in recent years. Genetic and experimental approaches have revealed a significant overlap between lipid storage disorders, most notably Gaucher’s disease, and synucleinopathies. However, does this and other pieces of evidence convince us that the key problem, the neurotoxicity-causing event, in synucleinopathies is the accumulation of (harmful) lipid species, leading to the phenomenon of “lipotoxicity”? What is the evidence, if any, that this kind of toxicity can be the consequence of, i.e., be downstream of, aberrant αS? In this article, we review the existing literature in an attempt to find an answer.

## 2. Synucleinopathies: Lipotoxicity in the Brain?

**Figure 1:**
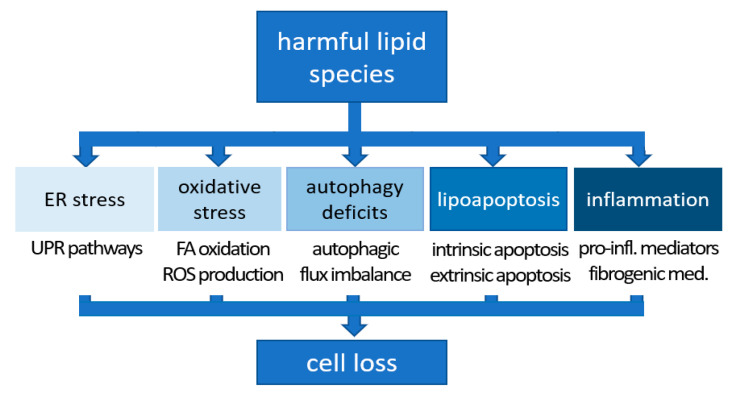
Lipotoxicity mechanisms. Lipid-excess-induced lipotoxicity causes ER stress, oxidative stress, autophagy impairment, lipoapoptosis and inflammation (adapted from [34] under a Creative Commons Attribution 4.0 International License: https://creativecommons.org/licenses/by/4.0/, accessed on 21 November 2021).

As mentioned above, a recent characterization of LBs by correlative light and electron microscopy (CLEM) came to surprising conclusions: fibrillar αS aggregates were found in about 20% of LBs and LNs examined, while abundant crowded organelles such as mitochondria, vesicle clusters and lipid membranes were detected in the core, which were coated with high concentration of non-fibrillar αS [15]. Another study, using label-free coherent anti-Stokes Raman scattering (CARS) microscopy found that the core of the LB lesions contained lipids in addition to proteins [16]. Outside LBs, C-terminally truncated αS-positive puncta localized at mitochondrial membranes in the cytoplasm [16]. Yet another study, which used the lipid dye boron-dipyrromethene (BODIPY) in histological stainings, concluded that dopaminergic neurons and midbrain microglia significantly accumulated intracellular lipids in PD substantia nigra (SN), while adjacent astrocytes had a reduced lipid load overall [18]. These studies, however, typically utilized material from sporadic PD patients and, therefore, there was no genetic insight into the relationship between αS imbalance and lipid alterations (upstream, downstream, unrelated?). αS accounts for as much as 1% of the total soluble protein content of the human brain while the lipid mass in the brain is second only to the adipose tissue [19]. It is well established that lipids account for approximately 10–12% of the fresh weight and half the dry matter of the brain [20]. For those reasons alone, it is not too far-fetched to think that the two, αS and lipids, are intimately linked. Moreover, it was noticed early on in the characterization of αS that it has resemblance with lipid-binding proteins [21]. The latter observation is consistent with a scenario in which (excess) αS binds to lipids [22,23,24,25] and to fatty acids (FAs) [26,27,28,29] thereby, e.g., triggering lipid changes via certain feedback mechanisms. In the following paragraphs, we will review studies from model organisms (paragraph 4) and human cells/tissues (paragraph 5) that are consistent with αS imbalance being upstream of detrimental lipid changes, i.e., “lipotoxicity”. Lipotoxicity commonly describes the stress that cells and organs experience upon prolonged exposure to excess lipids, triggering a flux into harmful pathways of non-oxidative metabolism [30,31,32]. The term “lipotoxicity” was first used to describe the functional deficits and type-2-diabetes-like phenotypes in pancreatic islets of rats overloaded with lipids [33]. Molecular mechanisms associated with lipotoxicity comprise oxidative stress, mitochondrial dysfunction, endoplasmic reticulum stress, autophagy impairment, and inflammation [31] (Figure 1; adapted from [34]). 

## 3. αS Imbalance Upstream of Lipid Alterations: Model Organisms

The clearest evidence of αS being upstream of (detrimental) lipid alterations can be found in one of the simplest model organism: αS expression in *Saccharomyces cerevisiae* (which does not naturally possess αS) leads to massive accumulation of lipid droplets (LDs) [35]. This finding was recently reproduced and studied in more detail: αS expression in yeast causes massive increases in triglycerides (TGs), diglycerides (DGs) and free FAs [36]. A lipid species specifically enriched in αS-expressing yeast is oleic acid (OA; 18 C-atoms, 1 double bond: 18:1) [36]. *Drosophila melanogaster,* another animal model that lacks endogenous αS, shows age-dependent locomotor dysfunction and neurodegeneration upon transgenic expression of human αS [37,38], thereby replicating key features of the human disorder. An unbiased proteomic analysis of αS expression in this system revealed significant enrichment of KEGG pathways that included FA biosynthesis (upregulated) and FA metabolism (downregulated) [39]. In a transgenic *Drosophila melanogaster* model of PD, in which human αS is specifically expressed in photoreceptor neurons, the accumulation of LDs induced by various LD proteins (perilipin1, perilipin2 or CG7900) was synergistically amplified by the co-expression of αS [40]. αS localized to LDs in both *Drosophila* photoreceptor neurons and in human neuroblastoma cells [40]. As far as vertebrate animal models are concerned (which naturally possess αS), observations from cultured mouse/rat neurons as well as mouse models have been reported. When rat cortical neurons were transduced with lentivirus expressing human wild-type (WT) αS and profiled by LC/MS at 14 and 20 days post transduction, the excess αS altered both neutral and phospholipids in a time- and dose-dependent manner. Neutral lipid elevations were most pronounced, and LD formation was increased [36]. A human fPD-linked E46K αS transgenic mouse model (displaying motor deficits) was shown to exhibit altered brain lipid homeostasis [36]. Brain levels of unsaturated FAs (UFAs), DGs, and TGs were significantly elevated in hE46K αS tg mice relative to hWT αS tg mice [36]. These in vivo data suggested that hE46K αS influences neutral lipid regulation and is associated with PD-relevant motor phenotypes. The αS ‘3K’ mouse model, based on a strategic amplification of hE46K (E35K+E46K+E61K), also features prominent lipid alterations [41]. More evidence of αS involvement in lipid regulation came from αS knock-out mice that showed altered polyunsaturated FA (PUFA) metabolism, with lower uptake of arachidonic acid in neurons/astrocytes and a decreased recycling from brain phospholipids [42,43,44,45,46,47]. In contrast, increased incorporation of docosahexaenoic acid in phospholipids and increased turnover were observed in the absence of *SNCA* [46]. The significance of these knock-out animal data relative to the putatively more disease-relevant overexpression models needs to be further explored. Nonetheless, all these findings from different model organisms combined highlight the role of αS in FA turnover and maintenance of lipid homeostasis. 

## 4. αS Imbalance Upstream of Lipid Alterations: Human Cells/Tissues/Body Fluids

The impact of αS on lipid metabolic pathways has also been studied in a number of human cell culture models. Altered FAs, in particular long-chain PUFAs such as 22:4 and 22:6, have been reported in mesencephalic neuronal cells that stably overexpress human αS as well as in PD/DLB brain lysates [48]. The authors observed increased membrane fluidity concomitant with increased αS expression. Thus, by modulating FAs, αS could theoretically impact diverse cellular functions such as receptor signaling, membrane transporter activation, ion channel conductance, and neurotransmitter release, since all of these are modulated by the membrane fluidity state. In addition, membranes play a vital role in the life cycle of neurotransmitters themselves, including storage and release from the synaptic vesicles, transport from the synaptic cleft, as well as their synthesis and degradation, which all could thus be also impacted [49]. This aspect has been described in detail [50,51]. When induced pluripotent stem cell (iPSC)-derived human neurons were lentivirally transduced to express αS, neutral and phospholipids were found to be altered, with the most notable change being increased TGs [36]. The TG buildup caused increased LD formation. FA profiling 14 days post transduction revealed increased OA in the WT αS-overexpressing neurons relative to vector control. Importantly, αS excess in iPSC-derived human neurons from αS triplication carriers demonstrated increased neutral lipid content [36]: human αS triplication and isogenic genetically corrected control lines were differentiated to neurons and profiled at 23 days in vitro. Lipid profiling identified the neutral lipid pathway to be altered by αS excess: αS triplication neurons exhibited increased DG relative to their genetically corrected controls [36]. Similarly, αS E46K lines (carrying fPD-linked αS E46K; [52]), when differentiated to neurons and profiled 36 days post terminal differentiation, revealed changes in the neutral lipid pathway: DG and TG levels were elevated relative to isogenic WT αS [36]. In another study, an overabundance of αS in cultured primary cortical neurons was shown to decrease lysosomal β-Glucocerebrosidase (GCase) activity, upregulating glucosylceramides and lysosomal stress in turn [53]. Encoded by the *GBA1* gene, the GCase enzyme catalyzes the conversion of glucosylceramide into glucose and ceramide [54]. Notably mutations in *GBA1* are the most common genetic risk factor for PD [55,56,57]. At least one study has reported elevated levels of glucosylceramide in the plasma of PD patients [58]. Lipidomic analyses of patient samples generally conclude that lipids are altered in PD (plasma, CSF, brains) relative to controls [59,60,61]. Wood et al., e.g., found by liquid-chromatography mass spectrometry that DAGs, lipid species typically associated with lipotoxicity (Figure 1), were significantly increased in the frontal cortex of PD patients [62]. The cohort with the most severe cortical neuropathology demonstrated the greatest elevation, again suggesting a potential link between αS and the neutral lipid pathway in PD etiology. We have recently summarized the outcome of many such studies on patient material in more detail [50,63], thereby also highlighting their variability which may be caused by different sample types, time points, sample treatments, PD stages, patient cohorts, and the rigor of the quantitative methods. Importantly, it should be noted that causality cannot be established from such studies. The main reason is that typically idiopathic PD was studied. Lipid changes in a cohort of αS mutant fPD patients (we are not aware of such a study) would be more informative in this regard, however, it would still be difficult to unequivocally characterize any lipid changes as the direct consequence of mutated αS and as a driver of toxicity. 

## 5. αS Imbalance Upstream of Lipid Alterations: Mechanistic Insight and Speculations

**Figure 2:**
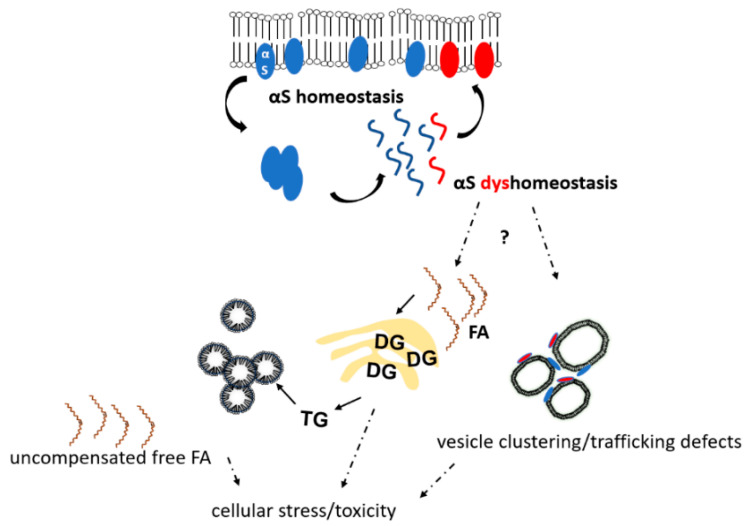
αS-induced lipid dyshomeostasis. αS homeostasis is characterized by balanced dynamic equilibria of soluble unfolded αS (blue wavy lines), membrane-associated αS monomers (blue ovals at membranes), and αS multimers (blue assembly in the cytosol). αS dyshomeostasis is initiated when αS monomers start to accumulate in the cytosol and/or at membranes (red wavy lines and ovals). By largely unknown mechanisms, αS dyshomeostasis triggers an imbalance in cellular lipid content, which comprises the accumulation of free FAs, DGs, TGs and oleic acid. αS excess at membranes (small red ovals, bottom right) causes vesicle clustering and/or trafficking defects. αS, α-synuclein; FA, fatty acid; DG, diacylglycerol; TG, triacylglycerol.

Cole et al. suggested that αS binds LDs and influences lipid metabolism by reducing the turnover of stored TGs within [64]. This mechanism—if relevant—would explain why LDs have been found to be elevated by experimentally increasing αS levels in several models (e.g., [36]). The high extent of LD increase, e.g., in yeast [35], would be consistent with feedback mechanism that increase lipid de novo generation when LD turnover is reduced by αS binding. In this regard it should be mentioned that a strategic amplification of αS E46K (‘3K’ = E35K+E46K+E61K) was found to be strongly associated with LDs in neural cells [65,66,67], and a similar trend was observed for E46K alone [65]. Strategic αS mutants that exhibit strongly enhanced LD binding [62,65,66] may be useful tools to elucidate signaling events that elevate (harmful) lipid species in experimental settings. It should be noted, however, that increased LDs in cells are not necessarily per se detrimental (their role as “friend or foe” was discussed in a recent review [68]). However, even if they shield cells from harmful effects of TG build-up by partitioning them [69], LDs may still serve as cellular stress indicators, highlighting the presence of more detrimental lipid species such as free FAs and DGs [70]. In support, interfering with TG/LD biosynthesis via diacylglycerol acyltransferases in yeast (deletion of DGAT orthologous genes) or in rodent cortical neurons (via depletion of DGAT1 and DGAT2) was reported to enhance αS toxicity [36,70]. In addition, a diacylglycerol kinase, DGKQ, that feeds into the DG pathway is a GWAS PD risk factor [71,72,73]. Mutations in DGKQ alter DG levels, which can result in trafficking defects and impact vesicle biology at synaptic terminals. Depending on the condition, the intracellular accumulation of detrimental lipid intermediates can cause cell death and cellular dysfunction in a variety of tissues including kidney, muscles, heart, and brain [74] (see also Section 1). While it is not fully understood how accumulating lipid intermediates directly contribute to cell dysfunction and death (Figure 1), it has been suggested that channeling free FAs into mitochondrial β-oxidation, structural lipids or LDs can mitigate harmful effects of excess lipid (reviewed in [68]). LDs are composed of a neutral lipid core that mainly contains TGs and cholesteryl esters surrounded by a phospholipid monolayer. Long perceived merely as inert fat reservoirs, LDs are now emerging as key organelles in lipid metabolism as well as essential for cellular stress response [75], and we expect LDs to gain more and more attention as key players in PD and other neurodegenerative diseases. The effect of αS dyshomeostasis on LDs/TGs/DGs and free FAs is not well understood, and Figure 2 summarizes the little insight we have and adds speculative aspects discussed in this paragraph (see legend for more details).

## 6. Diagnostic and Therapeutic Implications

Established biomarkers for PD or other synucleinopathies do not exist, and lipid-related biomarkers are no exception. Interesting in the context of this review is a recent study that highlighted lipid alterations in patient vs. control sebum, a non-invasively available biofluid [76]: using LC-MS, the authors report alterations in lipid metabolism related to the carnitine shuttle, sphingolipid metabolism, arachidonic acid metabolism and FA biosynthesis, which will require independent confirmation. There are many other lipidomic and metabolomic studies of patient postmortem brains, CSF and plasma samples (beyond the scope of this review and in part covered in our previous reviews [50,63]; see also Section 4). The majority finds lipids and FAs to be significantly different between PD patients and controls, but consensus is lacking, and it seems currently unclear if robust lipid biomarkers are within reach. More promising could be therapies that are based on the idea of restoring lipid homeostasis among neurons—but also astrocytes and microglia—to correct PD pathogenesis and slow disease progression [18]. A key strategy, moved forward by us and others, is the inhibition of stearoyl-CoA desaturase (SCD), a central player in lipid metabolism. SCD catalyzes the synthesis of monounsaturated FAs (MUFAs). MUFA are then incorporated into TGs and phospholipids. Consistent with αS triggering excess production of OA, DG and TG [36], inhibiting the rate-limiting enzyme in the biosynthesis of OA, SCD, was beneficial to αS-expressing human neural cells, rodent neurons and iPSC-derived neurons from αS triplication PD patients [36,77,78,79]. An unbiased compound screen in αS-expressing yeast also identified SCD as a therapeutic target that could neutralize the upregulation of MUFAs by excess αS [80]. Moreover, SCD inhibition has also been shown to improve symptoms in a mouse model that exhibits a PD-like, L-DOPA-responsive motor phenotype [81]. Therefore, there is considerable preclinical evidence suggesting that SCD inhibitors have the potential to ameliorate PD symptoms. Yumanity Therapeutics is currently testing an SCD inhibitor in clinical trials for PD [82]. The identification of SCD and the benefit observed by its inhibition strongly suggest FA dyshomeostasis/lipotoxicity imposed by αS can be ameliorated by modulating lipid pathways, and we expect other targets to emerge. The success of such treatments, however, is essentially unrelated to the question of whether lipid disturbance is downstream of, upstream of, or unrelated to αS imbalance, as long as lipid pathways contribute to pathogenesis and disease progression (see next section).

## 7. Concluding Remarks

**Figure 3:**
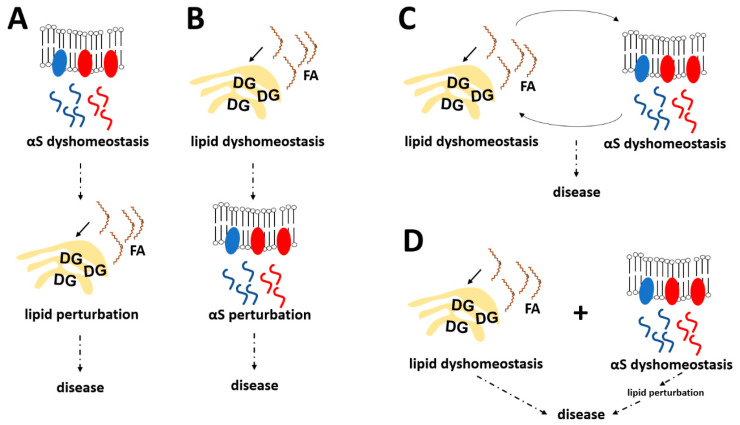
The interplay between αS and lipid biology in synucleinopathies—possible scenarios. (**A**) αS imbalance is upstream of abnormal lipid alterations. (**B**) Lipid imbalance is upstream of abnormal αS alterations. (**C**) αS imbalance and lipid imbalance influence and reinforce each other. (**D**) Primary lipid imbalance and αS-induced lipid imbalance are additive. αS, α-synuclein; FA, fatty acid; DG, diacylglycerol.

In this review we focused as much as possible on summarizing available evidence that αS dyshomeostasis can be unequivocally *upstream* of lipid alterations, which then become *drivers* of toxicity (“lipotoxicity”). Admittedly, it was not an easy task to ignore more complicated scenarios that, conversely, involve effects of lipids on αS. There are many indications that lipids also affect αS biology, not least since the discovery of lipid-related PD-risk genes such as *GBA* [55,56,57], *DGKQ* [71,72] and *ELOVL7* [83]. Thus, a hypothesis in which lipid disturbance is always downstream of αS in synucleinopathy (illustrated in Figure 3A) seems highly unlikely. However, the exact opposite hypothesis (αS dyshomeostasis always a consequence of lipid imbalance; Figure 3B) is not supported by the literature either, and our goal in this work was to highlight that there is ample evidence for αS alterations (increase, fPD mutations, engineered mutations) to cause detrimental lipid changes. Thus, it seems plausible that both “directions” contribute to disease, αS→lipid and lipid→αS complications are influencing each other, and the relative contribution of each may differ from case to case (Figure 3C). In this context, we have recently proposed a model where excess αS leads to an increase in OA levels, which in turn render αS more neurotoxic (and possibly more likely to elevate OA levels further), consistent with a vicious cycle [36,50,60]. Lowering the enzymatic formation of OA appears to benefit both cell health and αS biochemistry: it increased physiological αS multimerization, increased αS solubility, and decreased serine 129 phosphorylation [36,77]. In contrast, conditioning cells with MUFAs had the opposite effects [77]. These observations aligned in part with earlier work that demonstrated pathological αS oligomer accumulation by conditioning cultured neural cells with PUFAs [26]. 

Beyond neutral lipids, Mazzulli et al. have proposed a “bidirectional pathogenic loop” in which glucocerebroside catabolism affects αS levels, which in turn affect glucocerebroside catabolism. Related to this, inhibition of GCase activity in differentiated SH-SY5Y cells [84] or *GBA1* depletion/mutation in dopaminergic neurons differentiated from human PD-iPSCs with a heterozygous *GBA1* mutation (N370S/WT) led to accumulation of αS monomers [85]. Treatment with miglustat, a compound that reduces glucocerebroside synthesis, or augmentation of GCase protein reversed an increase in αS monomers and protected against PFF (pre-formed fibril)-induced human dopaminergic neuronal toxicity [85]. A role for GCase in lysosomal function has also been proposed, e.g., it was reported that a non-inhibitory small molecule modulator of GCase specifically enhanced activity within lysosomal compartments [78].

Together these data suggest bidirectional interplays between both αS-neutral lipid and αS-glucocerebroside/sphingolipid metabolism. To complicate things even further, neutral lipids and glucocerebroside/sphingolipid metabolism also influence each other. In this context, it was recently demonstrated that enhancing GCAse activity ameliorates both lipid disturbance and αS dyshomeostasis in PD mouse models [86]. It should be mentioned, however, that in a fourth scenario (Figure 3D), αS-induced and αS-unrelated lipid complications are simply additive, and idiopathic PD is triggered when a certain threshold of such complications is reached (aging-dependent lipid alterations may play a role). Last but not least, the toxic event in synucleinopathies (what kills the neurons?) remains unsettled, and given that the relevant literature is still emerging, it seems currently not possible to make a stronger case that “classical” lipotoxicity mechanisms (as described in Section 2 and Figure 1) are of key relevance in synucleinopathies. In addition, an increasing number of studies has demonstrated that αS aggregation and LB pathology can spread from neuron to neuron (presumably in a trans-synaptic fashion), which may even include proliferation from the peripheral nervous system to the brain [87,88]. The existence of multiple αS polymorphs or “strains” has been proposed to be responsible for the clinical heterogeneity among α-synucleinopathies [89,90]. In this context, it will be very interesting to learn if lipid abnormalities are upstream or downstream of pathogenic αS spread (or both), and if the heterogeneity in αS “strains” is paralleled by a heterogeneity in lipid alterations.

Overall, we are convinced that PD, LBD and MSA need to be treated as ‘lipidopathies’ as much as, if not more than, they have traditionally been considered to be ‘proteinopathies’ [18,50]. Future research will further elucidate in how far the classic understanding of lipotoxicity (Figure 1) applies to αS-triggered lipid changes in PD and other synucleinopathies. It seems possible that the concept of lipotoxicity may have to be modified to correctly describe the lipid alterations associated with cellular stress and cell loss in αS diseases (as in Figure 2).
